# Physiotherapy use is increased for up to nine months after receiving respiratory support for COVID-19

**DOI:** 10.1186/s12913-022-08870-x

**Published:** 2022-12-01

**Authors:** Katrine Damgaard Skyrud, Beate Margrethe Huseby, Karin Magnusson

**Affiliations:** 1 Norwegian Institute of Public Health, Cluster for Health Services Research, Postboks 222, Skøyen N-0213 Oslo, Norway; 2 Health Intelligence and Policy, The Norwegian Directorate of Health, Oslo, Norway; 3 Faculty of Medicine, Department of Clinical Sciences Lund, Orthopaedics, Clinical Epidemiology Unit, Lund University, Wigerthuset, Remissgatan 4, 22185 Lund, Sweden

**Keywords:** COVID-19, Respiratory tract infections, Physiotherapy, Respiratory support

## Abstract

**Aim:**

To explore whether physiotherapy use is increased after hospitalization with COVID-19 with or without respiratory support vs. other respiratory tract infections (RTI).

**Methods:**

In all Norwegian residents aged 18–80 years who were hospitalized with COVID-19 (*N* = 5,344) or other RTI (*N* = 82,235) between July 1st 2017 and August 1st 2021, we used a pre-post study design to explore the weekly individual average physiotherapy use in community care from 12 weeks prior to hospital admission, to 36 weeks (9 months) after hospital discharge for individuals who received and who did not receive respiratory support.

**Results:**

Prior to the hospital stay, COVID-19 patients and patients with other RTI had ~ 40–60 physiotherapist consultations per 1000 inpatients per week. COVID-19 patients on respiratory support had a higher increase in physiotherapy use after discharge than persons with other RTI on respiratory support (an additional 27.3 (95% confidence interval = 10.2 to 44.4) consultations per 1000 for men, and 41.8 (13.7 to 69.9) per 1000 for women)). The increase in physiotherapy use lasted for 6 months for men, and 9 months for women. COVID-19 inpatients without respiratory support had a similar up-to-9-months-change post-discharge physiotherapy use as inpatients with other RTI without respiratory support (-0.2 (-0.7 to 0.2) for men, and 0.09 (-6.4 to 6.6) for women).

**Conclusion:**

The need for physiotherapy was increased for up to 9 months after having COVID-19 requiring respiratory support vs. other RTI requiring respiratory support. No difference between diseases was seen for individuals who were hospitalized but not on respiratory support.

## Introduction

Persons who are admitted to the hospital with severe lung failure can report post-discharge long-term sequalae related to cognitive and lung functions [1, 2] A poor long-term prognosis may be expected for hospitalized COVID-19 patients (especially those who were more severely ill during their hospital stay), considering the affection of a range of bodily organs (for example, the lungs, heart, kidney, brain, muscles etc.) [3, 4]. There is also evidence that long-term mortality is increased because of long-term sequelae [5]. Because fatigue, reduced respiratory capacity, and muscle weakness have been reported to be prevalent in months after hospitalization with COVID-19 [3], rehabilitation services through physiotherapy have been incorporated into rehabilitation programmes in several countries [6, 7]. However, the actual and timely use, including demand and need for such services, are currently unknown.

Recent studies from the US, Germany, France and Finland reported higher in-hospital mortality, worse clinical outcomes and more severe complications among patients hospitalized for COVID-19 than patients hospitalized for influenza [8–10]. These studies, did not however, shed light on the consequences such worse outcomes would have for rehabilitation services, such as post-discharge physiotherapy. Also, there is very limited knowledge of the period such post-COVID-19 services are needed, compared to post severe influenza or other respiratory tract infections (RTI). Improved knowledge of how post-discharge physiotherapy use among men and women hospitalized with COVID-19 relates to post-discharge physiotherapy use among men and women hospitalized for other RTI, like influenza, would help the health services in planning for new waves of the pandemic.

We aimed to explore the use of physiotherapy services in community care from 12 weeks prior to admission to up to 36 weeks after discharge for all patients aged 18–80 year stratified by men and women, focusing on two comparisons: (1) Individuals with COVID-19 who *were* on respiratory support compared with individuals with other RTIs (influenza, upper and lower respiratory tract infections) who were on respiratory support, and (2) Individuals with COVID-19 who *were not* on respiratory support compared with individuals with other RTIs (influenza, upper and lower respiratory tract infections) who were not on respiratory support.

## Methods

### Design and data

We utilized population-wide registry data from the emergency preparedness register (BeredtC19) in Norway, which compiles daily updated individual-level data from several data sources. The register was established to give the Norwegian Institute of Public Health an ongoing overview and knowledge of the prevalence, causal relationships and consequences of the COVID-19 epidemic in Norway [11]. Data sources included in the current study comprised the Norway Control and Payment of Health Reimbursement (KUHR) Database (physiotherapy visits), the Norwegian Patient Registry (NPR) (data on hospitalization, with dates and causes) and the National Population Register (data on age, sex, death and emigration). All data were collected retrospectively, yet we applied a prospective study design, including a pre-post comparison of individuals with COVID-19 vs. with other RTI who were vs. who were not on respiratory support during their hospital stay. The emergency preparedness register was established as part of the legally mandated responsibilities of The Norwegian Institute of Public Health (NIPH).

### Study population

We studied adults between 18 and 80 years old who were hospitalized between July 1st 2017 and August 1st 2021 and who could be observed for at least 24 weeks before the day of hospital admission and at least 24 weeks after the day of discharge (meaning that they did not have a cause for which they could not be observed, for example turning 81 during this period). Elderly (80 years or more) were excluded as they are less often hospitalized and instead may receive treatment in care facilities (of which the data are not available). Non-residents such as tourists and temporary workers were excluded. The earliest possible start of an individual’s pre-period fell on the date lying 24 weeks prior to July 1st 2017, and the latest possible end of post-period fell on 24 weeks after August 1st 2021. The long duration of the inclusion period was due to the very few other circulating viruses alongside the SARS-CoV-2 virus in 2020-21, giving very few hospitalizations due to other RTI in these years. Thus, to have data allowing for comparison of COVID-19 to other RTI, we included earlier seasons back to 2017. Patients who died or emigrated after their discharge were censored from the study at date death or emigration, whichever came first. The terms admission and discharge are used to describe the first and last day of a hospitalization spell. Hospitalization spells with any mention of the included diagnostic codes that occurred with less than two calendar days in between, were coded as one hospitalization spell, i.e., with the same admission ID and date of admission and discharge.

We categorised all hospitalized patients into two mutually exclusive diagnosis categories with a primary/secondary diagnosis code registered in NPR as:

- COVID-19 (ICD-10 codes: U071, U072) or.
- other RTI including influenza (ICD-10 codes: J0, J09, J10, J11, J13, J14, J15, J16, J17, J18, J20, J21, J22, J80).

A washout period of 6 months (24 weeks) was used to only include new incident cases of one of the diagnosis categories. Patients who were re-admitted to hospital and thereby not eligible to use physiotherapy were censored during their hospital stay.

We studied COVID-19 and other RTIs with and without a need for respiratory support, including records of both invasive procedure codes (GXAV01) and non-invasive procedure codes (GXAV10, GXAV20, GXAV23 and GXAV30) according to the Norwegian version of the Nordic Medico-Statistical Committee (NOMESCO) Classification of Medical Procedure (NCSP-N) [12].

### Outcomes

We studied visits/consultations to the physiotherapist in primary care/community services from 12 weeks prior to hospital admission, to 36 weeks after discharged from hospital, for patients diagnosed with COVID-19 and other RTI. The length of pre- and post-periods were chosen based on data availability and decisions made in our previous post-covid research [13, 14]. Because the study subjects were more severely ill with a hypothesized longer impact on the need for healthcare services after the illness, we included a longer post-discharge period in the current study than in previous studies (36 weeks, as compared to 24 weeks). In Norway, physiotherapists are either publicly employed (most often by the municipalities) or they have private practices. Most private practitioners have contracts with the municipalities which gives reimbursement rights (from the state) and some basic funding from the municipalities. Other private practices are all private and charge the patient’s full price. In the current study, we included both private (with operating agreement in the form of a reimbursement contract) and public physiotherapists. Physiotherapist with a reimbursement contract constituted about 60% of physiotherapy man-years in primary care [15]. Physiotherapists without such contracts were not available in the data.

### Statistical analyses

First, we studied the proportion of the included individuals visiting the physiotherapist at least once per week from 12 weeks prior to hospitalisation, to 36 weeks after discharged from hospital after COVID-19 vs. after other RTI.

Second, we estimated the difference in change of physiotherapy use over time for our four study groups (men and women with or with respiratory support), using a difference-in-difference (DiD) approach [14]. In short, the DiD approach evaluates the effect of an event by comparing the change in the outcome in the pre-period with the post-period. Hence, we compared the rate of physiotherapy visits in the months before hospitalisation and after discharge for both groups. The DiD estimate is calculated by comparing the differences (change in the outcome before and after hospitalization) for the various groups. Statistically, we included an interaction term between the dependent variable (physiotherapy visits) and the kind of the disease (COVID-19 or other RTI). We specifically studied the group difference in physiotherapy use for five post-discharge periods: 1–4 weeks, 5–12 weeks, 13–20 weeks, 21–28 weeks and 29–36 weeks.

Since several confounding variables may affect the baseline probability of using physiotherapy, we adjusted for age (18–29, 30–64 and 65–80 years), country of birth (Norway or abroad) and comorbidities (0, 1, 2, or 3 or more) defined as risk factors for COVID-19 by an expert panel [16]. We included calendar month fixed effects to account for background trends like seasonal variation in physiotherapy use and vaccination rates. In addition, we added an interaction between all covariates and time (relative week) to account for time-varying nature of the confounder and obtain an unbiased result [17].

Data management and statistical analyses were run in R version 4.0.2.

## Results

**Fig. 1:**
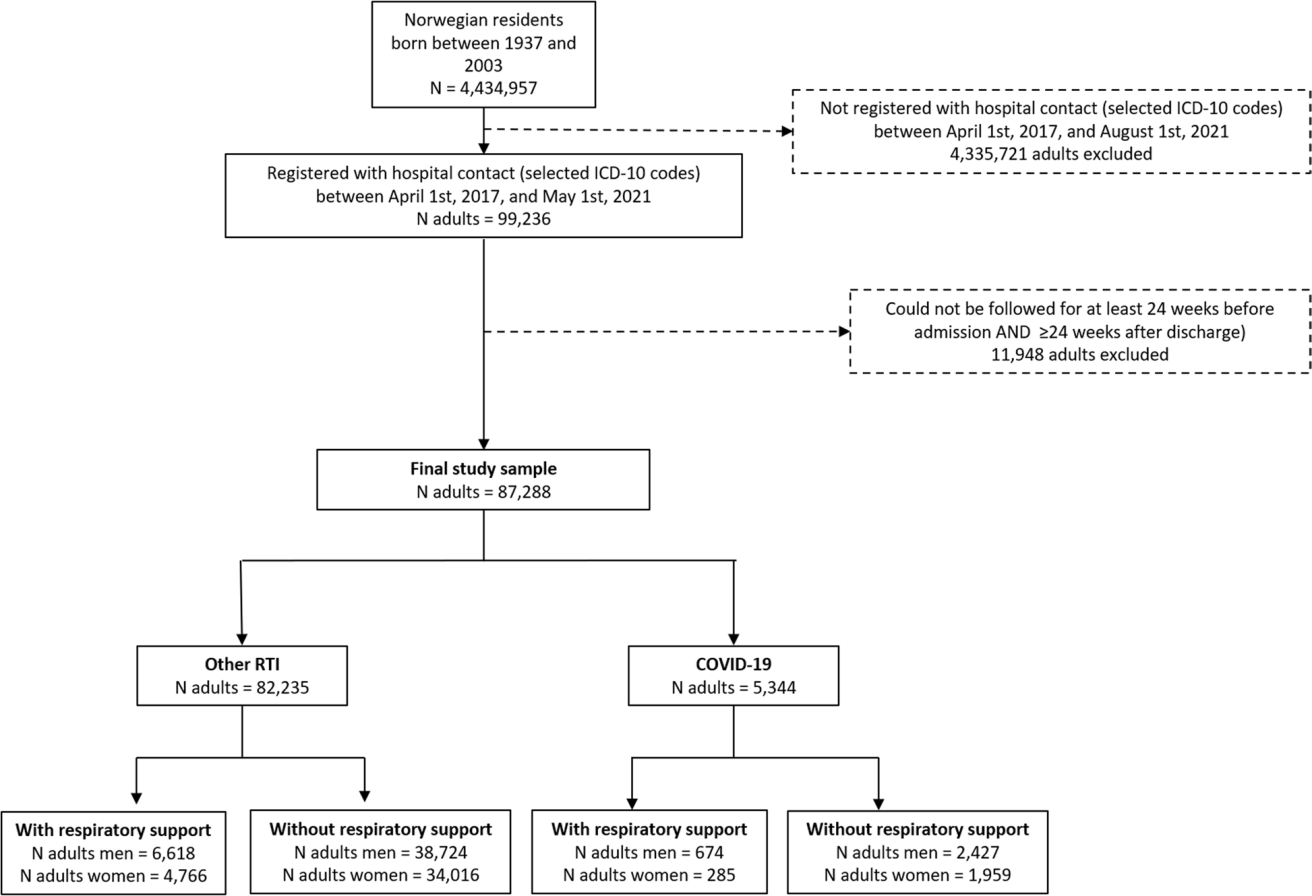
Flowchart of the study population

Of about 4.4 million (*n* = 4 434 957) Norwegian residents aged 18–80 years, 99 236 had been registered with hospital contact for COVID-19 or other RTI diagnosis between April 1st, 2017, and May 1st, 2021; 11 948 were excluded because they could not be followed for at least 24 weeks before admission and 24 weeks after discharge (Fig. 1). We studied in total 87,288 persons (18–80 years) who were hospitalized with COVID-19 (*N* = 5,344) and/or other RTI (*N* = 82,235) between July 1st 2017 and August 1st, 2021 (Fig. 1). More men than women were hospitalized with COVID-19 (58,1% men) and other RTI (61,3% men). Men and women suffering from COVID-19 (with or without respiratory support) were generally younger, had less comorbidities, were more often born abroad, and had longer hospital stay (only for those in need of respiratory support), compared to men and women with other RTI (Table 1). In addition, men hospitalized with COVID-19 were more often on respiratory support during their hospital stay (674 of 3,104 = 21.7%) than women (285 of 2,249 = 12.7%) (Table 1). In comparison, only 13.6% of men and 11.8% women hospitalized with other RTI received respiratory support (Table 1). Influenza patients constituted about 6% (with respiratory support) and 10% (without respiratory support) in men and 8% (with respiratory support) and 11% (without respiratory support) in women for patients with other RTI (Table 1).

**Table 1:** Descriptive characteristics

	Men		Women	
	Hospitalized with COVID-19	Hospitalized with other RTI	Hospitalized with COVID-19	Hospitalized with other RTI
**With respiratory support**				
Adults, n	674	6,618	285	4,766
Hospitalizations, n	674	6,868	285	5,049
Length of stay in days, mean (SD)	22.7 (18.8)	18.8 (22.9)	22.4 (17.8)	15.8 (19.5)
Age, mean (SD)	58.1 (12.3)	63.2 (14.0)	59.3 (12.3)	64.0 (13.5)
Born abroad, n (%)	306 (45.4)	634 (9.2)	134 (47.0)	342 (6.8)
Two or more comorbidities, n (%)	111 (16.5)	2,021 (29.4)	59 (20.7)	1,539 (30.5)
Influenza, n (%)	N/A	434 (6.3)	N/A	404 (8.0)
**Without respiratory support**				
Adults, n	2,427	38,724	1,959	34,016
Hospitalizations, n	2,430	43,564	1,964	37,717
Length of stay in days, mean (SD)	5.6 (9.0)	6.1 (8.1)	4.9 (7.1)	5.2 (6.6)
Age, mean (SD)	53.9 (14.8)	61.0 (16.4)	50.7 (16.1)	57.9 (18.1)
Born abroad, n (%)	1,059 (43.6)	4,746 (10.9)	885 (45.1)	3,905 (10.4)
Two or more comorbidities, n (%)	378 (15.6)	13,134 (30.3)	262 (13.3)	9,603 (25.5)
Influenza, n (%)	N/A	4,218 (9.7)	N/A	4,133 (11.0)

**Fig. 2:**
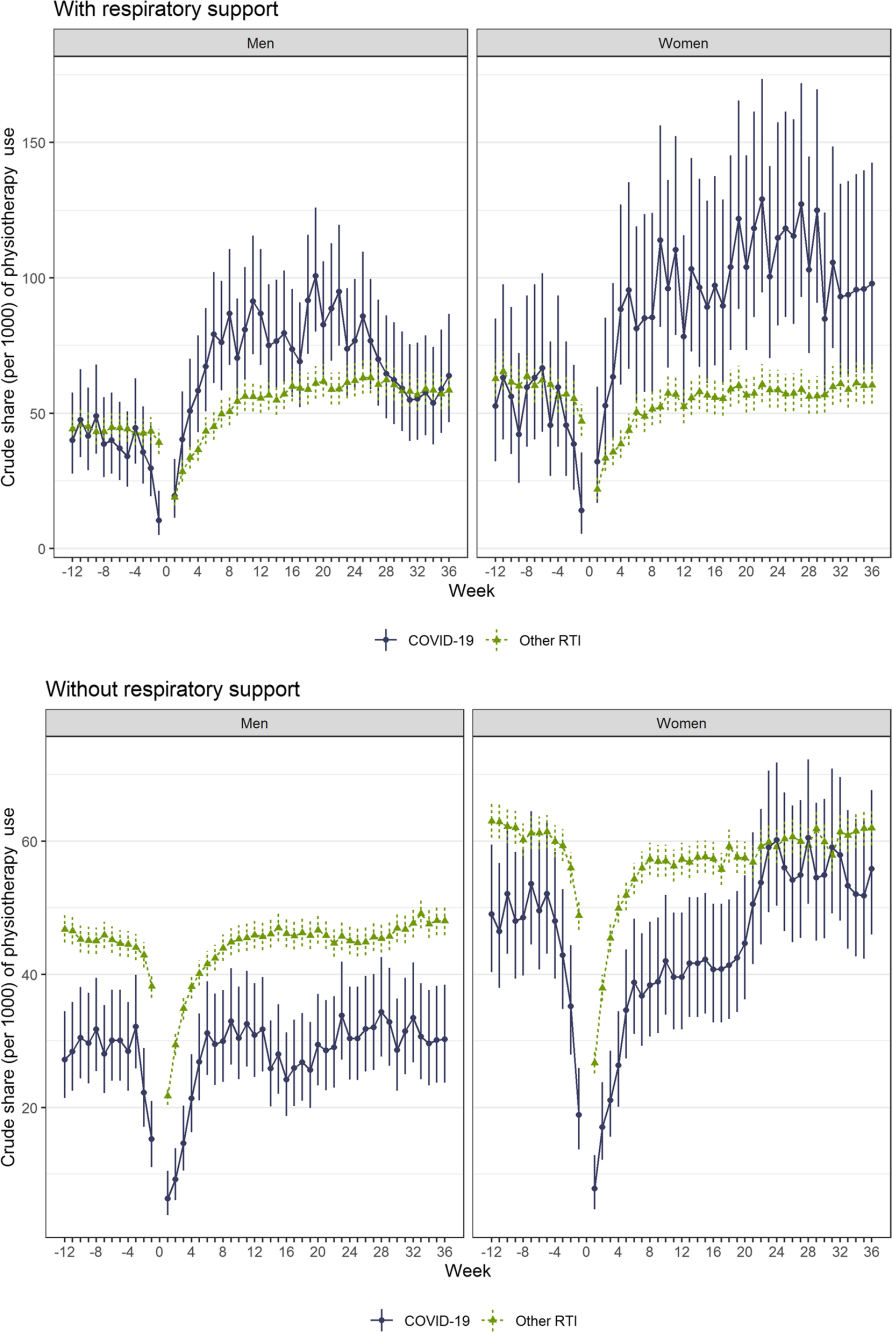
Crude share (per 1000) and 95% CI of all-cause physiotherapy use per week, from 12 weeks before hospitalization to 36 weeks after hospitalization, for COVID-19 patients and other RTI patients, separately for men and women with and without respiratory support

About 41 (95% CI = 36 to 47) per 1000 of the male patients with COVID-19 who received respiratory support visited a physiotherapist at least once per week during the 12 − 5 weeks before admission to hospital, dropping markedly in the 4 weeks before admission (Fig. 2, top left panel). Similarly, about 45 (95% CI = 43 to 47) per 1000 of other RTI male patients who received respiratory support visited a physiotherapist 12 − 5 weeks before hospitalization (Fig. 2, top left). For women with respiratory support, about 56 (95% CI = 47 to 66) per 1000 patients with COVID-19 and about 62 (95% CI = 60 to 65) per 1000 patients with other RTI visited a physiotherapist at least once per week during the 12 − 5 weeks before admission to hospital (Fig. 2, top right). Lower pre-levels of physiotherapy use were found for men and women patients with other RTI without need for respiratory support (~ 30 per 1000 for men and 50 per 1000 for women). For all four groups, the share using physiotherapy gradually increased from 1 to 4 weeks after discharge.

For post-discharge levels of physiotherapy use from 5 weeks and onwards, there was an important within-group difference. COVID-19 inpatients on respiratory support had a higher share visiting the physiotherapist at least once per week compared to inpatients with other RTI on respiratory support (with between 1 and 36 weeks an average of 71 (95% CI = 68 to 74) per 1000 male patients and 97 (91 to 103) per 1000 female patients) (Fig. 2, top panels). In contrast, patients without need for respiratory support with COVID-19 and other RTI had the same pre- and post-discharge shares visiting the physiotherapist at least once per week after than before discharge, except for the sudden increase from 20 to 24 weeks for women with COVID-19 (Fig. 2, bottom panels).

When statistically comparing the pre- and post-discharge periods for the four study groups using the differences-in-differences method, we found elevated risk for both men and women with respiratory support. For men with COVID-19 on respiratory support, we found elevated use of physiotherapy for COVID-19 patients up to 28 weeks when compared to patients with other RTI (with respiratory support) (Table 2). The overall elevated use of physiotherapy for the whole period between 1 and 36 weeks after discharges was β of 27.3 (95%CI = 10.2 to 44.4) per 1000 for men with COVID-19 on respiratory support. For women with COVID-19 on respiratory support we found elevated use of physiotherapy throughout the period (β of 41.8 (95%CI = 13.7 to 69.9) per 1000, more specifically with average weekly number of physiotherapy visits per 1000 of 32.3 (95%CI = 3.2 to 63.3) between week 1 and 4 to 36.6 (95%CI = 0.8 to 72.4) between week 29 to 36 (Table 2). No elevated use of physiotherapy was found for COVID-19 patients without respiratory support when compared to patient with other RTI without respiratory support, with average weekly number of physiotherapy visits per 1000 of -0.2 (95%CI= -0.7 to 0.2) between week 1 and 36 for men, and 0.09 (95%CI= -6.4 to 6.6) for women.

**Table 2:** Impacts of being hospitalized with COVID-19 on physiotherapy use in adults, using adults hospitalized with other RTI as comparison group, separately for men and women with and without respiratory support

	With respiratory support				Whitout respiratory support			
	Men		Women		Men		Women	
	β	95 % CI	β	95 % CI	β	95 % CI	β	95 % CI
1-4 weeks	19.1	2.7 - 36.0	32.3	3.2 - 61.3	-3.3	-8.0 - 1.3	-8.4	-15.5 - -1.2
5-12 weeks	38.8	18.9 - 58.8	43.1	11.8 - 74.3	1.1	-4.2 - 6.3	-3.2	-10.1 - 3.6
13-20 weeks	38.6	18.21 - 59.1	43.5	10.4 - 76.6	-3.0	-8.6 - 2.7	-0.2	-7.7 - 7.2
21-28 weeks	23.6	1.5 - 45.6	47.9	11.8 - 84.1	-3.6	-10.1 - 2.8	4.4	-4.4 - 13.2
29-36 weeks	8.5	-13.5 - 30.4	36.6	0.8 - 72.4	-4.9	-11.6 - 1.7	4.0	-5.2 - 13.1

## Discussion

### Principal findings

In 87,288 patients hospitalized with COVID-19 or other RTI in Norway between July 1st 2017 and August 1st 2021, we found a six to nine months increased physiotherapy use among COVID-19 inpatients on respiratory support relative to inpatients on respiratory support for other RTI. Inpatients with COVID-19 and RTI not in need of respiratory support had lower and similar levels of physiotherapy from before to after their hospital stay.

### Comparison with previous studies

To our knowledge this is the first study to examine the post-covid use of physiotherapy for patients who are severely affected by the initial disease. Thus, we found no studies for an effective comparison of our findings. Previous studies of healthcare use following mild and severe COVID-19 have been focused towards visits to the general practitioner and specialists [13, 14, 18, 19], which may not reflect the need for rehabilitation services. In that regard, the use of physiotherapy may serve as a proxy for the need, but also as a proxy for the offers of physiotherapy rehabilitation. The current study is also one of very few that has compared COVID-19 outcomes with comparable diseases, like influenza, supporting the findings from recent studies from the US, Germany, France and Finland, where a higher ventilator (respiratory support) use and longer hospital stay were related to worse clinical outcomes such as more frequently developing acute respiratory distress syndrome, pulmonary embolism, septic shock, or haemorrhagic stroke [8–10]. Moreover, we demonstrate important sex differences. Because men had a far higher share of hospitalized patients on respiratory support than women (19.4% vs. 10.8%) (Table 1), the need for physiotherapy may be higher among men than women after hospitalization with COVID-19. These findings are in line with other studies stating that the men are more severely affected by COVID-19 than the women [20, 21].

### Clinical implications

Our findings have several implications for policy makers, for medical staff (and most importantly for physiotherapists) as well as for the understanding of disease severity in terms of post-covid rehabilitation need. First, our findings may imply that the community physiotherapy services might need to be upscaled in periods of high transmission with SARS-CoV-2 variants causing severe disease. Second, physiotherapists need to be prepared as to how to meet patients that have been hospitalized and on respiratory support following their discharge. For example, physiotherapists may need to learn how to optimally evaluate and treat severely ill COVID-19 patients when they present with long-term complaints at the clinic. We suggest that the already started initiatives aiming to find the most optimal physiotherapy rehabilitation for COVID-19 and initiatives aiming to form guidelines for the clinical management of COVID-19 continue with similar force [22, 23]. A final implication of our findings is that physiotherapists might need to focus more on the most severely affected COVID-19 patients, i.e. those requiring respiratory support. Besides the increased need for post-covid physiotherapy use after COVID-19 requiring respiratory support, an important finding of our study was that COVID-19 patients not requiring respiratory support had a similar prognosis in terms of physiotherapy use as patients with other RTI not requiring respiratory support. The latter finding implies that moderately severe COVID-19 (requiring hospitalization but not e.g. ventilation) does not require more from physiotherapists than moderately severe other RTI (requiring hospitalization but not e.g. ventilation). Considering that the currently dominating SARS-CoV-2 omicron variant (with or without previous vaccination) leads to milder disease [24], our findings may imply that physiotherapists will not be overloaded by hospital-admitted COVID-19 patients in the future. Still, new and more serious variants may be arise [25], and our findings underlines the need for physiotherapists to be prepared.

### Strengths and limitations

An important strength of this study is the inclusion of a comparison group that consists of patients who are hospitalized for comparable severe disease, i.e. other RTI including influenza. Given that the two are respiratory diseases with similar modes of transmission and same kind of seasonality, we could put the burden of severe COVID-19 on the community physiotherapy services in the context. Other important strengths are the use of routinely collected data and the use of modern methods for causal inference, like the differences-in-differences methods.

Some limitations should be mentioned. First, we could only include physiotherapists with a reimbursement contract in our study, meaning that physiotherapists on hospitals or at private institutions are not included. There is often long waiting time for treatment for physiotherapist with a reimbursement contract and especially for physiotherapists with experience with treating patients with fatigue etc. It is therefore possible that the COVID-19 patients, who can be hypothesized to have more fatigue [3], more often were sent to a private rehabilitation institution if their post-discharge complaints were worse, which prevented them from using their community physiotherapy services (and hence, prevented them from being registered in our data) during their rehabilitation stay. Such skewed use of physiotherapy that is registered vs. not registered in our data across exposure groups might have led to an underestimation of the effect of COVID-19 on physiotherapy use when compared to other RTI. However, to date, such rehabilitation at institutions has been challenging to get access to, supporting the notion that most COVID-19 and other RTI patients get their rehabilitation services in the community health services. Differential access to different types of physiotherapy might explain the sudden increase in physiotherapy use seen for COVID-19 patients who were not on respiratory support (Fig. 2). However, the increase was not statistically significant as compared to weeks 1–20 (Fig. 2). A second limitation may be that factors related to selection could have impacted on our findings. As an example, we know that COVID-19 hit immigrant groups in Norway particularly hard [26, 27], which might not be the case for other RTI or influenza. The lower percentage of patients visiting physiotherapist per week among COVID-19 patients compared to other RTI may be due to the fact that the COVID-19 patient were younger and had less comorbidities.

## Conclusion

We found a six to nine months increased physiotherapy use among COVID-19 inpatients on respiratory support relative to inpatients on respiratory support for other RTI. Because physiotherapists can expect to meet severely ill COVID-19 patients for a long time after their hospital stay, our study demonstrates the need for physiotherapists to find optimal rehabilitation strategies for this growing patient group.

## Data Availability

The individual-level data used in this study are not publicly available due to privacy laws, but the data in the registries compiled in Beredt C19 are accessible to authorized researchers after ethical approval and application to helsedata.no administered by the Norwegian Directorate of eHealth. Data are however available from the authors (Katrine Skyrud) upon reasonable request and with permission of Norwegian Directorate of Public health and Norwegian Institute of Public Health.
